# Feasibility and safety of whole-body vibration therapy in intensive care patients

**DOI:** 10.1186/s13054-017-1669-2

**Published:** 2017-04-13

**Authors:** Peter Alter, Tobias Boeselt, Christoph Nell, Marc Spielmanns, Klaus Kenn, A. Rembert Koczulla

**Affiliations:** 1 Department of Medicine, Pulmonary and Critical Care Medicine, University Medical Center Giessen and Marburg, Philipps University Marburg, Baldingerstrasse, 35033 Marburg, Germany; 2 Outpatient Pulmonary Rehabilitation, Remigius Hospital, 51379 Leverkusen-Opladen; Medical School, University of Witten/Herdecke, 58448 Witten, Germany; 3 Department of Respiratory Medicine and Pulmonary Rehabilitation, Schoen Klinik Berchtesgadener Land, 83471 Schoenau am Koenigssee; Philipps University Marburg, Baldingerstrasse, 35033 Marburg, Germany

We read with great interest the article by Wollersheim and colleagues who examined whole-body vibration (WBV) in intensive care unit patients [1]. Hemodynamic characteristics were monitored during WBV application. The study underscores previous findings showing that no significant changes in heart rate, blood pressure, or oxygen saturation occurred during WBV in critically ill patients, nor when compared with healthy controls [2].

In the present study, unconscious sedated patients received WBV in a flat supine position without any changes in body position, except flexion of the hips and knees. The method raises the question whether enough load was applied to the vibrating plate to lead to a sufficient neuromuscular response. To increase this load and involve neuromuscular recruiting, it might be helpful to modify the patient’s position by inclination of the bed to approximately 20° to 25° degrees of tilt [2]. It is suggested that this would involve a greater muscular proportion of the whole body. In conscious patients, additional training effects may be achieved by the use of a (yet customized) vibrating dumbbell for the upper extremities.

Since the recent method is preliminary in the current setting, potential effects on muscle function and morphology should be assessed in further studies [3]. A short-term response could be detected, e.g., by electromyography [2]. Longer-term effects involve muscular morphology, e.g., hypertrophy, which can be assessed by sonography-based morphometry [4].

It remains debatable whether catecholamines influenced the findings in the present study, since no controls were examined [5].

In summary, WBV, if applicable in conjunction with a vibrating dumbbell, appears safe and feasible in early rehabilitation. Potential beneficial long-term effects remain to be shown.

## Abbreviations

WBV: Whole-body vibration
